# Tumor-specific hepatic stellate cells (tHSCs) induces DIgR2 expression in dendritic cells to inhibit T cells

**DOI:** 10.18632/oncotarget.19027

**Published:** 2017-07-05

**Authors:** Yun-Hong Xia, Zhen Lu, Min Zhao, Wen-Ting Dai, Lu Ding, Li-Xia Hu, Guo-Lin Jiang

**Affiliations:** ^1^ Department of Oncology, The Fourth Affiliated Hospital, Anhui Medical University, Hefei 230022, China; ^2^ Department of General Surgery, The Fourth Affiliated Hospital, Anhui Medical University, Hefei 230022, China; ^3^ Hefei Hospital, Anhui Medical University, Hefei 230011, China; ^4^ Key Laboratory of Anhui Medical University, Hefei 230061, China

**Keywords:** hepatic stellate cells (tHSCs), hepatocellular carcinoma (HCC), DIgR2, dendritic cells, tumor immunity

## Abstract

Tumor-specific hepatic stellate cells (tHSCs) contributes to tumorigenesis and progression of hepatocellular carcinoma (HCC). The potential function of tHSCs on dendritic cells (DCs) was studied here. We discovered that tHSCs co-culture induced upregulation of DIgR2 (dendritic cell-derived immunoglobulin receptor 2) in bone marrow-derived DCs (mDCs). Activation of MEK-ERK is required for DIgR2 expression in mDCs. MEK-ERK inhibitors or shRNA-mediated silence of MEK1/2 in mDCs inhibited tHSCs-induced DIgR2 expression. Meanwhile, tHSCs stimulation decreased production of multiple cytokines (CD80, CD86 and IL-12) in mDCs. Such an effect was almost reversed by DIgR2 shRNA in mDCs. Further, tHSCs-stimulated mDCs induced T-cell hypo-responsiveness, leading to decreased cytotoxic T lymphocyte (CTL) activity and reduced IFN-γ production in splenic T cells. T cell proliferation inhibition and apoptosis were also noticed. These actions on T cells were again largely inhibited by DIgR2 shRNA in mDCs. Together, our results indicate that tHSCs directly induces DIgR2 expression in DCs to inhibit T cells.

## INTRODUCTION

Hepatocellular carcinoma (HCC) is the fifth most common cancer worldwide [1–3]. The prognosis of HCC has been poor, especially for the patients with advanced and/or metastatic tumors [4–6]. Recent epidemiological data indicates that HCC's mortality rate could possibly be doubled over the next decades [4–6].

Hepatic stellate cells (HSC) are the main source of extracellular matrix proteins during fibrogenesis [7–9]. Recent studies have proposed that tumor-specific hepatic stellate cells (tHSCs) are critical in the HCC tumorigenesis and progression [7–9]. These cells, characteristically expressing α-smooth muscle actin (α-SMA), could possibly proliferate and differentiate into myofibroblasts [7–9]. tHSCs are tumor-promoting cells, which express intercellular adhesion molecule 1 (ICAM-1) and vascular cell adhesion molecule 1 (VCAM-1) among other factors to promote cancer cell migration and proliferation [7–9].

Recently studies, including ours [10, 11], have also proposed a key function of tHSCs in tumor immunology [7–9]. We have previously shown that tHSCs could induce T cell apoptosis [10]. Further, tHSCs also inhibits T cell response, causing HCC cell migration and invasion [11]. Dendritic cells (DCs) are specialized antigen-presenting cells (APCs), which are responsible for the initiation and regulation of immune response [12, 13]. Activation of DCs is important for anti-tumor activity [12, 13]. Dendritic cell-derived immunoglobulin receptor 2, or DIgR2, is a key inhibitory receptor that inhibits DCs-induced antigen-specific T-cell responses [14]. DC-derived DIgR2 is shown to bind to the receptor in T cells, suppressing T-cell proliferation, cytokine production and cytotoxic T lymphocyte (CTL) activity [14]. In the current study, we show that tHSCs induces DIgR2 expression in DCs, causing T-cell hypo-responsiveness.

## RESULTS

### tHSCs co-culture induces DIgR2 expression in bone marrow-derived dendritic cells (mDCs)

**Figure 1:**
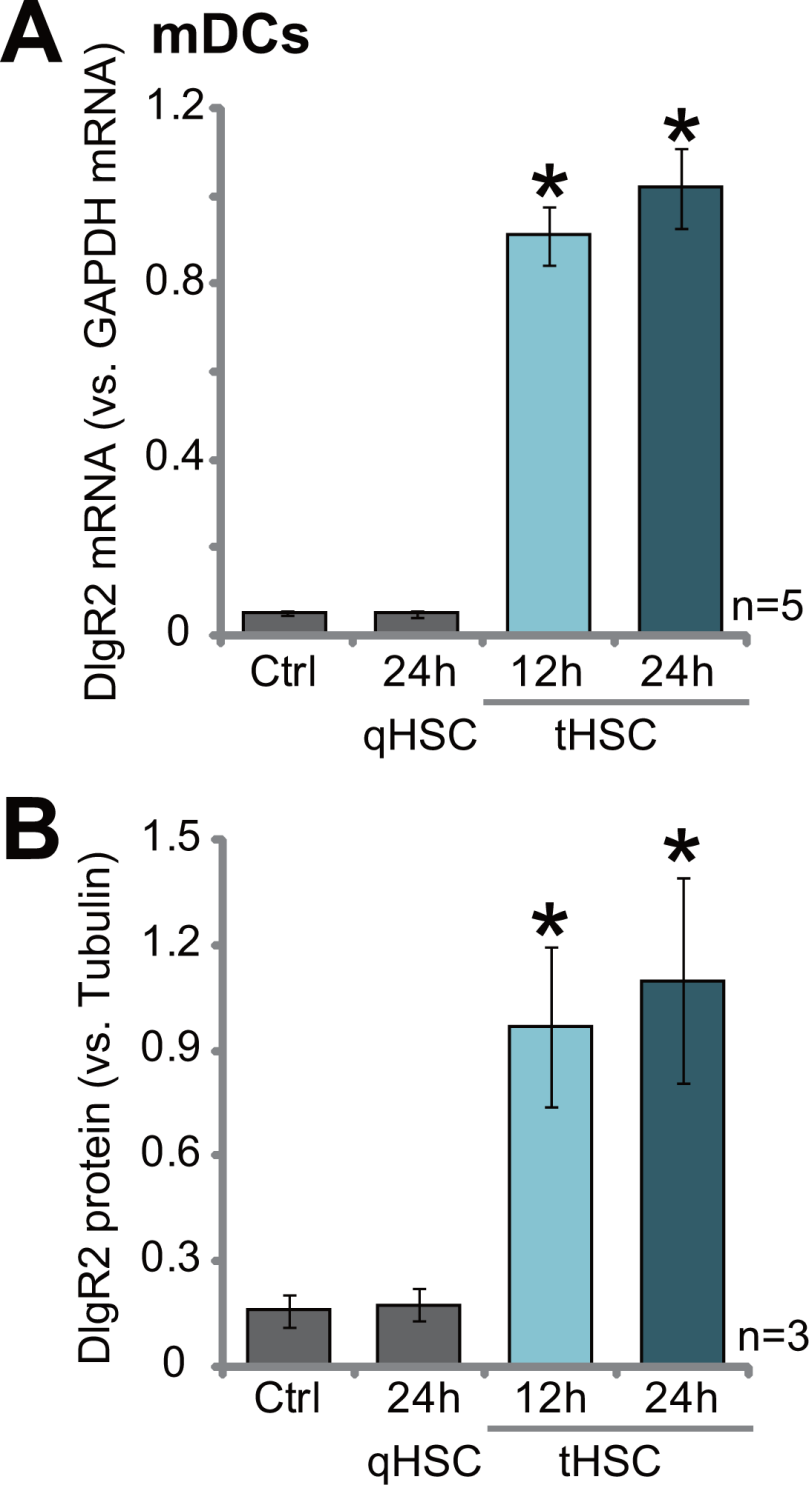
tHSCs co-culture induces DIgR2 expression in bone marrow-derived dendritic cells Relative *mRNA* (**A**) and protein expression (Three sets of repeated blot data were quantified in (**B**) of DIgR2 in bone marrow-derived dendritic cells (mDCs), co-cultured with/out quiescent HSCs (qHSCs) or tumor HSCs (tHSCs) for applied time, were shown. “Ctrl” stands for mDCs only. “Tubulin” stands for loading control β-Tubulin. *P < 0.05 vs. “Ctrl” group.

As described in our previous studies [10], the primary HSCs were derived from livers of control normal Buffalo rats or the xenograft HCC tissues, which were named as quiescent HSCs (qHSCs) and tumor HSCs (tHSCs), respectively [10]. Primary cultured bone marrow-derived dendritic cells (mDCs) were also derived from Buffalo rats [14]. mDCs were then co-cultured with tHSCs or qHSCs (mDCs to HSCs ratio, 20: 1). Expression of DIgR2, the key immune inhibitory receptor of immunoglobulin superfamily [14], was then tested. Quantitative real-time PCR (“qRT-PCR”) assay results in Figure 1A demonstrated that *DIgR2* *mRNA* expression in mDCs was significantly elevated after co-culture of tHSCs. *DIgR2* *mRNA* level increased over 9–10 fold higher in mDCs with tHSCs challenge (Figure 1A). On the other hand, qHSCs co-culture had no significant effect on *DIgR2mRNA* expression (Figure 1A). Further, DIgR2 protein expression in mDCs was also dramatically induced when co-cultured with tHSCs (but not the qHSCs, three sets of repeated data were quantified in Figure 1B). These results suggest that DIgR2 expression is induced in mDCs after tHSCs co-culture.

### MEK-ERK activation is required for DIgR2 expression in tHSCs-stimulated mDCs

**Figure 2:**
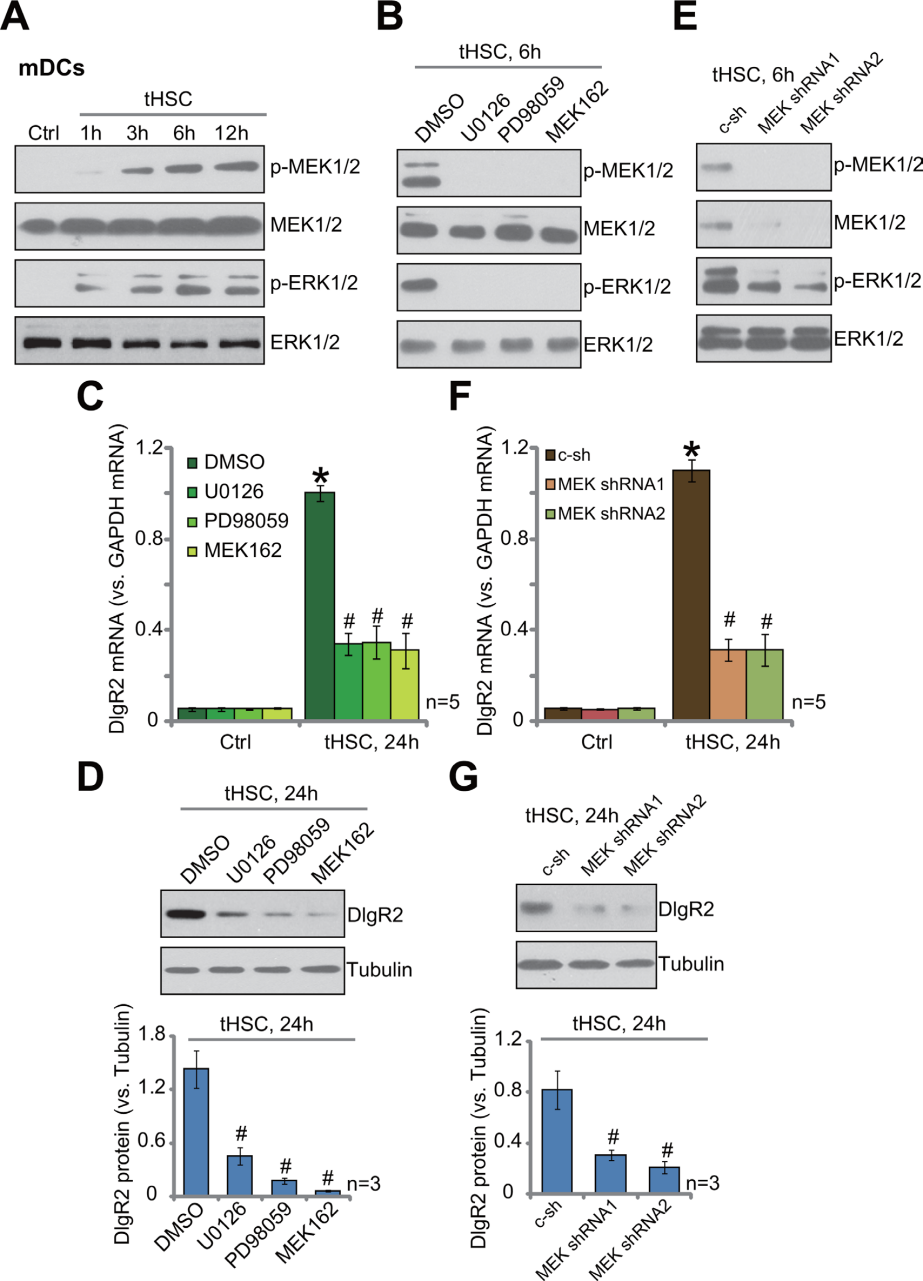
MEK-ERK activation is required for DIgR2 expression in tHSCs-stimulated mDCs Bone marrow-derived dendritic cells (mDCs) were co-cultured with tumor HSCs (tHSCs) for indicated time; MEK-ERK signaling activation was tested by Western blotting assay (**A**). mDCs were pretreated with PD98059 (100 nM), U0126 (100 nM), MEK162 (1 μM) or vehicle (0.1% “DMSO”) for 1 hour, followed by tHSCs co-culture for applied time; Signaling was tested by Western blotting assay (**B**); DIgR2 *mRNA* (**C**) and protein (**D**), three sets of repeated blot data were quantified) expressions were also tested. mDCs were infected with applied MEK1/2-shRNA (“MEK shRNA1/2”) or non-sense scramble control shRNA (“c-sh”), cells were further co-cultured with tumor HSCs (tHSCs) for indicated time; Signaling was tested by Western blotting assay (**E**); Relative mRNA (**F**) and protein expression (G, three sets of repeated blot data were quantified) of DIgR2 were also tested. “Ctrl” stands for mDCs only. *P < 0.05 vs. “Ctrl”. ^#^*P* < 0.05 vs. “DMSO” group (C) or “c-sh” group (D, F and **G**).

Next, we studied the potential mechanism of DIgR2 expression in mDCs with tHSCs co-culture. Western blotting assay results showed that co-culture with tHSCs induced significant activation of MAPK/ERK kinase (MEK)-extracellular signal-regulated kinase (ERK) cascade in mDCs (Figure 2A). Phosphorylated (p-) MEK1/2 and p-ERK1/2 in mDCs were significantly increased following co-culture of tHSCs (Figure 2A). To study the link between MEK-ERK activation and DIgR2 expression, pharmacological MEK-ERK inhibitors were first applied, including PD98059, U0126 and MEK-162 [15, 16]. As shown in Figure 2B, treatment with these inhibitors almost completely blocked MEK-ERK cascade activation in mDCs with tHSCs co-culture. Consequently, DIgR2 *mRNA* (Figure 2C) and protein (Figure 2D) expressions were also largely inhibited. The pharmacological evidences suggest that activation of MEK-ERK signaling is required for DIgR2 expression in tHSCs-stimulated mDCs.

To further support our hypothesis, shRNA method was applied to knockdown MEK1/2 in mDCs. Two MEK1/2 shRNAs with non-overlapping sequences, named as “MEK shRNA1/2”, were applied. MEK1/2 expression was indeed dramatically downregulated after shRNA infection (Figure 2E). MEK-ERK activation in mDCs, tested against by p-MEK1/2 and p-ERK1/2, was also largely attenuated (Figure 2E). tHSCs-stimulated DIgR2 *mRNA*(Figure 2F) and protein (Figure 2G) expression was largely inhibited in MEK-silenced mDCs. Collectively, these results suggest that activation of MEK-ERK cascade is required for DIgR2 expression in tHSCs-stimulated mDCs.

### shRNA knockdown of DIgR2 in mDCs

**Figure 3:**
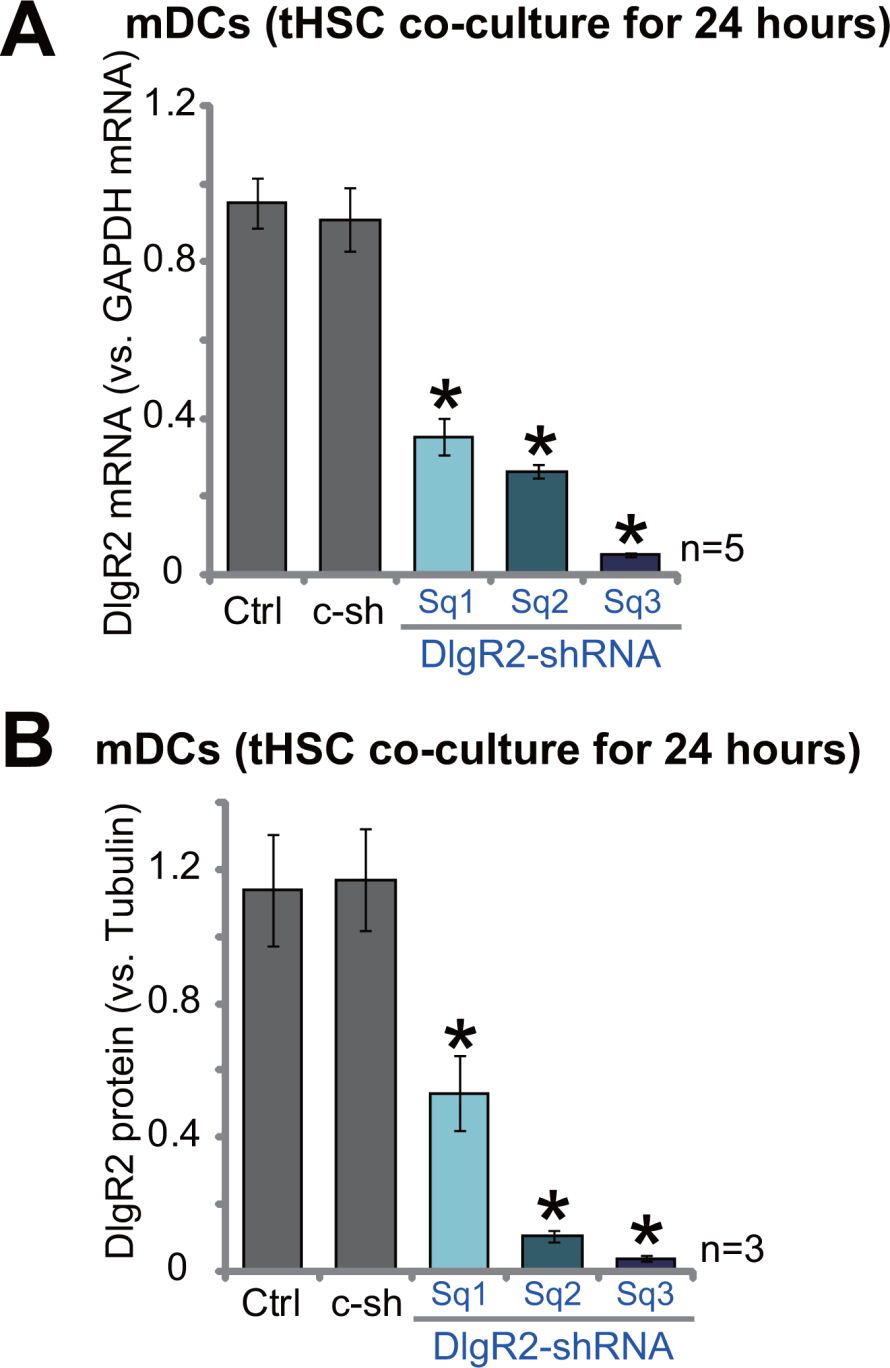
shRNA knockdown of DIgR2 in bone marrow-derived dendritic cells (mDCs) Bone marrow-derived dendritic cells (mDCs) were infected with DlgR2-shRNA (“Sq1/2/3”) or non-sense scramble control shRNA (“c-sh”), cells were further co-cultured with tumor HSCs (tHSCs) for 24 hours, relative *mRNA* (**A**) and protein expression (Three sets of repeated blot data were quantified in (**B**) of DIgR2 were shown. “Ctrl” stands for no shRNA group. *P < 0.05 vs. “Ctrl”.

The aim of this study is to test the potential effect of tHSCs on the activity of DCs. More specifically, we wanted to know if tHSCs-induced DIgR2 expression could influence the functions of mDCs. Thus, lentiviral shRNA method was applied to selectively knockdown DIgR2 in mDCs. A set of three lentiviral DIgR2 shRNAs, targeting the non-overlapping sequence of DIgR2, were established. The three shRNAs were named as “DlgR2-shRNA Sq1/2/3”. qRT-PCR assay results in Figure 3A confirmed that adding each of the three shRNAs significantly inhibited *DIgR2 mRNA* expression in mDCs with co-culture of tHSCs. Consequently, DIgR2 protein expression was also silenced (Three sets of repeated data were quantified in Figure 3B). Among three tested DIgR2 shRNAs, the DIgR2 shRNA “Sq3” showed highest efficiency in knocking down DIgR2 (Figure 3A and 3B). This DlgR2-shRNA (“Sq3”) was then selected for further functional studies. Notably, the non-sense scramble control shRNA (“c-sh”) failed to decrease DIgR2 expression in mDCs (Figure 3A and 3B). Notably, DIgR2 shRNA failed to inhibit survival of mDCs (tested by the trypan blue assay).

### tHSCs co-culture inhibits production of multiple cytokines in mDCs, abolished after DlgR2 silence

**Figure 4:**
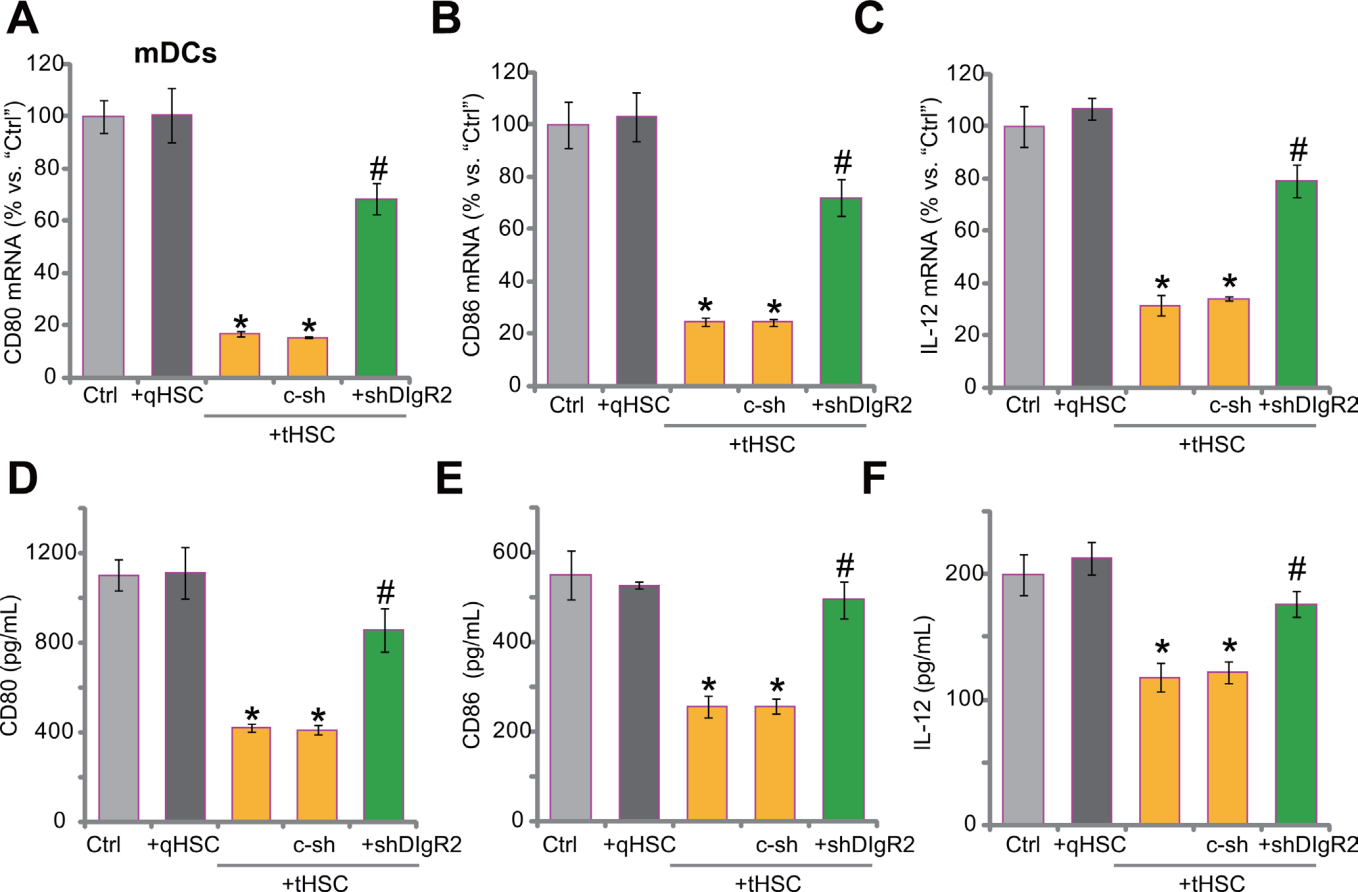
tHSCs co-culture inhibits production of multiple cytokines in mDCs, abolished after DlgR2 silence mDCs were infected with DlgR2-shRNA (“shDlgR2, Sq/3”) or non-sense scramble control shRNA (“c-sh”), cells were further co-cultured with tumor HSCs (tHSCs) for 72 hours, *mRNA expression* (in mDCs, qRT-PCR assay, (**A–C**) and protein content (in conditional medium, ELISA assay, (**D–F**) of listed cytokines were analyzed. “Ctrl” stands for mDCs only. *P < 0.05 vs. “Ctrl”. ^#^*P* < 0.05 vs. “c-sh” group

Next, we tested the function of mDCs after co-culture of tHSCs. Several DC-associated cytokines or activation markers, including CD80, CD86 and interleukin (IL)-12 [17–20], were tested. qRT-PCR assay was performed, and results showed that *mRNA* expressions of CD80 (surface co-stimulatory molecule, Figure 4A), CD86 (another surface co-stimulatory molecule, Figure 4B) and IL-12 (DC activation marker cytokine, Figure 4C) in mDCs were decreased significantly after co-culture of tHSCs. On the other hand, qHSCs co-culture failed to change *mRNA* expression of the above-mentioned cytokines (Figure 4A–4C). Remarkably, DlgR2 silence, by the DlgR2-shRNA (“Sq3”) in mDCs, almost restored *mRNA* expression of the cytokines (Figure 4A–4C). These results indicate that DlgR2 induction by tHSCs is important for subsequent inhibition of above cytokine expression. Further ELISA assay results showed that productions of CD80 (Figure 4D), CD86 (Figure 4E) and IL-12 (Figure 4F) were also decreased in tHSCs-stimulated mDCs. Such effects were again almost abolished with shRNA knockdown of DlgR2 (Figure 4D–4E). Notably, the non-sense scramble control shRNA (“c-sh”) was in-effective (Figure 4A–4F).

### tHSCs-stimulated DlgR2 expression in mDCs inhibits splenic T cells

**Figure 5:**
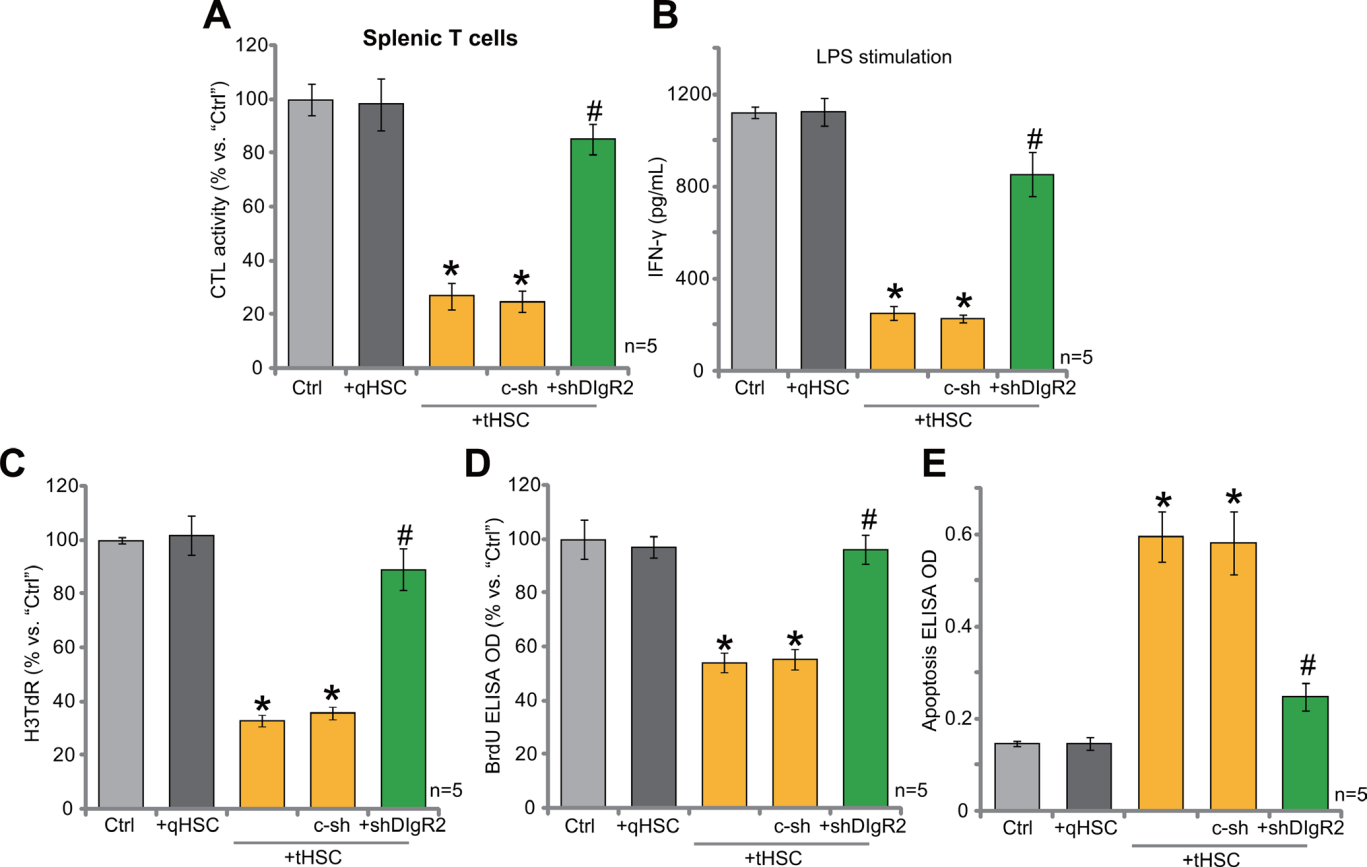
tHSCs-stimulated DlgR2 expression in mDCs inhibits splenic T cells mDCs were infected with DlgR2-shRNA (“shDlgR2, Sq/3”) or non-sense scramble control shRNA (“c-sh”), cells were further co-cultured with/out tumor HSCs (tHSCs) or quiescent HSCs (qHSCs) for 24 hours; Stimulated mDCs were then co-cultured with splenic T cells; OVA-II peptide CTL assay activity (after 24 hours, (**A**), LPS (100 ng/mL)-induced IFN-γ production (after 24 hours, (**B**), cell proliferation (**C** and **D**), after 96 hours) and cell apoptosis (**E**), Histone DNA ELISA assay, after 96 hours) in the T cells were analyzed. “Ctrl” stands for co-culture of regular mDCs. *P < 0.05 vs. “Ctrl”. *P < 0.05 vs. “c-sh”.

It has been previously shown that DC-derived DIgR2 binds to the receptor in T cells, which shall inhibit normal T cell functions. Thus, we then co-cultured splenic T cells with mDCs (T cells/mDCs ration, 20:1). OVA-II peptide CTL assay results in Figure 5A confirmed that co-culture with tHSCs-stimulated mDCs significantly inhibited the CTL activity of splenic T cells. Further, lipopolysaccharide (LPS)-induced interferon γ (IFN-γ) production in splenic T cells was also largely inhibited with the existence of tHSCs-stimulated mDCs (Figure 5B). Remarkably, such effects in splenic T cells were almost completely reversed with silence of DlgR2 in tHSCs-stimulated mDCs (Figure 5A and 5B). These results indicate that tHSCs-stimulated DlgR2 expression/production in mDCs should mediate inhibition of splenic T cells. Further studies confirmed that co-culture of tHSCs-stimulated mDCs induced proliferation inhibition (Figure 5C and 5D) and apoptosis (Figure 5E) in splenic T cells, which were again largely attenuated with DlgR2 shRNA in mDCs (Figure 5C–5E). Notably, qHSCs-stimulated mDCs had no significant inhibition on splenic T cells (Figure 5A–5E). Also, the non-sense scramble control shRNA (“c-sh”) failed to rescue splenic T cells from tHSCs-treated mDCs (Figure 5A–5E).

## DISCUSSION

tHSCs are the major source of extracellular matrix proteins during fibrogenesis [21–23], and are vital in the tumorigenesis and progression of human HCC [7, 8]. tHSCs could infiltrate HCC stroma and peri-tumor tissues, and could also be localized around tumor sinusoids, fibrous septae and the tumor capsule [7–9]. tHSCs promote HCC progression via regulating diverse biological processes, *i.e*. facilitating extracellular matrix (ECM) turnover, growth factor and cytokine signalling, as well as promoting tumor angiogenesis [7–9]. Recent studies have also proposed a pivotal function of tHSCs in tumor immunity [7–9].

Immune evasion of HCC cells [24–26] and other tumor cells is critical for cancer progression [26, 27]. One underling mechanism is to decrease the number and activity of anti-tumor immune cells, at both the tumor site and in the lymphoid organs [26, 27]. tHSCs could induce T-cell hypo-responsiveness, an effect that is not observed with the qHSCs. Previous studies have shown that B7-H1 (a key member of the B7 family of co-stimulatory molecules) upregulation in tHSCs could result in increased ligation of PD-1 receptor on activated T-cells, causing T cell apoptosis and inhibition of T-cell-mediated tumor cell apoptosis [28–30]. Meanwhile, activated tHSCs could also increase the number of in T-reg cells to exert immunosuppressive functions [31, 32].

We here discovered another key mechanism of tHSCs in immuno-regulation: via direct communicating with DCs. tHSCs stimulation induced upregulation of DIgR2 in mDCs, which is a key negative regulator of DC-initiated T-cell responses [14]. Further, tHSCs-stimulated mDCs induced T-cell hypo-responsiveness, causing decreased CTL activity and reduced IFN-γ production as well as T cell proliferation inhibition and apoptosis. Remarkably, such effects were largely attenuated with DIgR2 shRNA in mDCs. Based on these results, we propose that tHSCs stimulation directly induces DIgR2 expression and production in DCs, which binds to T-cells, causing T-cell hypo-responsiveness and apoptosis.

Intriguingly, we showed that activation of MEK-ERK cascade is required for tHSCs-stimulated DIgR2 expression in mDCs. MEK-ERK inhibitors (PD98059, U0126 and MEK-162) or shRNA-mediated knockdown of MEK1/2 largely attenuated tHSCs-induced DIgR2 expression in mDCs. Further studies will be needed to further characterize the underlying signaling mechanisms of DIgR2 expression in mDCs.

## MATERIALS AND METHODS

### Chemicals, reagents and antibodies

All the antibodies utilized in this study were obtained from Abcam (Suzhou, China). The cell culture reagents were obtained from Invitrogen (Shanghai, China). MEK-ERK inhibitors, PD98059, U0126 and MEK-162 [33, 34], were provided by Selleck (Beijing, China). The mRNA primers were designed and synthesized by the Genepharm (Shanghai, China).

### Rat HCC tumor model

The detailed protocol of rat tumor model was described in detail in our previous studies [10, 11]. Briefly, buffalo rats (4–5 week-old) were obtained from the Animal Center of Soochow University (Suzhou, China). The MRH HCC cells [10, 11] were injected into the right flanks of the Buffalo rats. Four weeks after the initial injection, xenograft tumors were established. Surgery-isolated fresh HCC tumors were then cut into small piece (2×1×1 mm^3^), and were transplanted to the rat livers [10], using the described procedures [10]. Tumor extension was then determined by ultrasound on days 7, 14, 21 and 28 after tumor establishment. The animal protocols were approved by Institutional Animal Care and Use Committee (IACUC) and Ethics Board of Anhui Medical University. All surgical procedures were performed with anesthesia. All efforts were made to minimize suffering.

### Isolation and culture of HSCs

As described in our previous studies [10, 11], HSCs were derived from the livers of normal Buffalo rats or HCC tissues. The livers were subjected to perfusion with described solution [10, 11]. Afterwards, truncated normal liver tissues and HCC tissues were further digested using the described method [10, 11]. Thereafter, the cell suspensions were established, which were purified by centrifugation through a 8% Nycodenz (Axis-Shield PoC) gradient. The achieved HSCs were then cultured in DMEM medium (GIBCO-BRL) with 10% FBS. Cell viability over 90% was verified via trypan blue exclusion. The desmin immuno-staining assay was performed to determine the purity of quiescent HSCs (qHSCs) and tHSCs, ranging from 90–95% [35].

### Primary culture of bone marrow dendritic cells (mDCs)

As described [10, 36], bone marrow were isolated from Buffalo rat femurs. After lysis of the red blood cell, bone marrow cells were cultured in described RPMI-1640 medium [10, 11] with rat recombinant granulocyte-macrophage colony stimulating factor (GM-CSF, 4 ng/mL, Sigma) and interleukin-4, (IL-4, 1,000 U/mL, R&D systems, Shanghai, China). The non-adherent cells were released spontaneously from the proliferating cell clusters, harvested, washed, and resuspended in the described complete medium [36].

### mDCs and HSC co-culture

For each well, 2.5 × 10^5^ mDCs were incubated with 1.25 × 10^4^ HSCs (20: 1, mDCs to HSCs) for applied time.

### Primary culture of spleen T cells

As described in our previous studies [10, 11], the minced rat spleens were filtered. The splenocytes were then isolated from erythrocytes via centrifugation of the cell suspension on a Ficoll gradient (Histopaque 1083, Sigma) [37]. The cells were layered onto the top of the gradient in a 10-ml Falcon tube, followed by centrifugation at 800×g for 20 min at room temperature without braking. Lymphocytes (mainly T-cells) were collected, and washed twice in PBS-1% FBS (washing buffer). Cells were then cultured.

### Co-culture of spleen T cells with mDCs and *in vitro* T cell function detection

The spleen T cells were utilized as antigen-specific responders and co-cultured with mDCs (T cells/mDCs ratio = 20:1). OVA (323–339) peptide assay was performed to test CTL assay [14]. T cell proliferation was determined by [^3^H]-TdR DNA incorporation assay (after 96 hours [14]) and BrdU ELISA assay (after 96 hours [38]) using the attached protocols. Histone DNA apoptosis ELISA kit was applied to test cell apoptosis (after 96 hours), and detailed protocol was described previously [39–41]. T cells were also treated with LPS (100 ng/mL, Sigma), supernatants were collected after 24 hours for ELISA detection of IFN-γ ( R&D Systems).

### RNA extraction and real-time PCR

RNA was always extracted by the TRIzol reagents (Promega, Shanghai, China) [42]. cDNA was achieved via reverse transcription under the SYBR Green PCR kit (Applied Biosystems, Suzhou, China). Quantitative real time-PCR (qRT-PCR) assay was performed via the ABI Prism 7600 Fast Real-Time PCR system as previously described [43, 44]. Melt curve analysis was applied to calculate product melting temperature. *Glyceraldehyde-3-phosphatedehydrogenase* (*GAPDH)* mRNA was chosen as the reference gene, and the 2^−∆ ∆Ct^ method was utilized to quantify targeted mRNA change within samples [43, 44]. *DIgR2 mRNA* primers were described previously [14]. *GAPDH primers* were also described [43, 44].

### Western blotting assay

Cell lysates (30–40 μg each condition) were separated by the 10–12% Sodium Dodecyl Sulphate Poly Acrylamide Gel Electrophoresis (SDS-PAGE) gels, which were transferred to the polyvinylidene difluoride (PVDF) membranes (Millipore, Suzhou, China) [45]. The blots were blocked (with 5% non-fat milk) and incubated with designated primary plus secondary antibodies. Enhanced chemiluminescence (ECL, Roche, Shanghai, China) detection kit was utilized to visualize the targeted band/s under x-ray films. The intensity (total gray) of each band was quantified via ImageJ software, and was normalized to the loading control (β-Tubulin) [40].

### Enzyme-linked immunosorbent assay (ELISA) assay

Cytokine production was examined by the corresponding ELISA kit (R&D Systems), and detailed protocol was described previously [46].

### DIgR2 shRNA

Three distinct lentiviral DIgR2 shRNAs (named as “Sq1/2/3”) were packed into the GV248-puromycin vector, and were provided by Genepharm (Shanghai, China). The lentiviral shRNA (10 μL/mL per well) was added directly to mDCs for 24 hours. DIgR2 expression in the resulting cells was tested by qRT-PCR assay and Western blotting assay. DIgR2 shRNA “Sq3”, with targeted sequence of AAGAAGAGCTGGTGAACAAC, showed highest efficiency in downregulating DIgR2.

### MEK1/2 shRNA

Two lentiviral MEK1/2 shRNAs were also provided by Genepharm (Shanghai, China), which were also added to cultured mDCs for 24 hours. MEK1/2 expression in the resulting cells was tested by Western blotting assay.

### Statistical analysis

All statistical analyses were conducted using SPSS 15.0 software. Values were expressed as the mean ± standard deviation (SD). *P* < 0.05 was considered to indicate a statistically significant result.

## CONCLUSIONS

These results indicate that tHSCs directly induces DIgR2 expression in DCs to inhibit T cells.
